# Analysis and Differences of the Neurosurgical Complications During COVID-19 Pandemic

**DOI:** 10.14744/SEMB.2021.87120

**Published:** 2021-12-29

**Authors:** Balkan Sahin, Mustafa Kilic, Levent Aydin, Saime Ayca Sahin, Baris Ozoner, Enes Akkaya, Adem Yilmaz, Ahmet Murat Musluman

**Affiliations:** 1. Department of Neurosurgery, University of Health Sciences Turkey, Sisli Hamidiye Etfal Training and Research Hospital, Istanbul, Turkey; 2. Department of Neurosurgery, University of Health Sciences, Cam ve Sakura Training and Research Hospital, Istanbul, Turkey; 3. Department of Neurosurgery, Bahcesehir University Faculty of Medicine, Istanbul, Turkey

**Keywords:** Complications, coronavirus disease, neurosurgical operations, pandemic

## Abstract

**Objectives:**

The objective of the study was to analyze the complications of neurosurgical operations during the COVID-19 pandemic by comparing them with the complications observed in the pre-pandemic period.

**Methods:**

Two groups were formed: (1) Patients who were operated in the 5-month period of the pandemic (March–July 2020) and (2) those who were operated the same operations in the same period 1 year before (March–July 2019). Demographics, characteristics, medical follow-up data, complications, and outcome compared between the groups.

**Results:**

Similar demographics were observed between the groups. The number of all neurosurgical cases and neurotrauma cases decreased by 79% and 68% in pandemic period, respectively. The rate of emergency surgeries was significantly higher in pandemic group (p<0.001). The operation time was significantly longer in pandemic group (p=0.014). Total complication rate was significantly higher in pandemic group (p=0.002). Specifically, the rate of pulmonary complications was significantly higher during pandemic period (p<0.001). The infection rate (p<0.001), antibiotic use (p<0.001), and intensive care unit stay (p=0.023) were significantly higher in pandemic group.

**Conclusion:**

During pandemic period complication rates increased and a higher risk than expected was encountered. Treatment should be performed by taking the precautions and informing the patients about additional risks.

Coronavirus disease 2019 (COVID-19) has emerged as a life-threatening and extremely infectious condition. The World Health Organization (WHO) declared COVID-19 as an international public health emergency on January 30, 2020.^[1]^ COVID-19, which has spread rapidly after its first detection in China in December 2019, has affected more than 170 million cases worldwide and caused more than 3.7 million deaths.^[2]^ Mandatory revisions have been made in all health-care systems within the scope of combating the COVID-19 pandemic. In this context, the WHO has published guidelines, including the increasing need for healthcare services and the increasing need for intensive care units (ICUs) to prepare for the fight against the pandemic. ^[3]^ Based on the nature of pandemics, healthcare workers are in the highest risk group. Consistent with this information, healthcare workers accounted for approximately 10% of positive cases in Italy and China.^[4]^ In this pandemic that strains the capacities of healthcare professionals and systems, the whole world, and health-care systems are going through a tough test. In neurosurgical procedures, pre-operative and post-operative patient follow-up and meeting the need for intensive care are vital.^[5]^ In a period when existing workforce and resources are primarily devoted to combating the pandemic, neurosurgical practice is inevitably negatively affected. Thus, this study compares the effects of COVID-19 between March 2019 to July 2019 and March 2020 to July 2020 by comparing patients who underwent similar surgeries and complications. Moreover, the results of this study could help fight the problems we may encounter in neurosurgical practice in the pandemic that is likely to recur.

## Methods

### Neurosurgery in the COVID-19 Pandemic

Various guidelines have been published to regulate the surgical processes to be applied to neurosurgery patients during the COVID-19 pandemic. In our clinic, care was taken to apply the guidelines of the Turkish Ministry of Health, European Association of Neurosurgical Societies, American Association of Neurological Surgeons, and Turkish Neurosurgical Society, and in line with these guidelines, each patient was evaluated individually, and surgical scheduling was made.^[6]^ Surgery was recommended in cases in which surgical intervention would not lead to irreversible effects (Table 1). For example, surgery was not planned for patients with asymptomatic benign or low-grade tumors and spinal degenerative and peripheral nerve diseases without neurological deficits. Surgery was planned if the patient would be harmed if the surgery was delayed, for example, patients with primary malignant brain tumors, metastasis, any cranial and spinal lesion causing progressive neurological deficit, trauma, or vascular disease complicated with hemorrhage.

**Table 1.** Proposal chart for scheduling developed using recommendations from the European association of neurosurgical societies, American association of neurological surgeons, British neurosurgical society, and Turkish neurosurgical society^[6]^

	**Low-acuity surgery Proposal; postponing surgery**	**Intermediate-acuity surgery Proposal; postponing surgery if possible**	**High-acuity surgery Proposal; performing the surgery without postponing**
Neurovascular	Cranial nerves		
	Microvascular decompression	Unruptured aneurysm	Ruptured/unstable aneurysm
		Arteriovenous malformation	Arteriovenous malformation hemorrhage
			Intracerebral hematoma (depend on the space occupied)
			Malignant cerebral artery infarction
Neuro-oncology	Asymptomatic benign tumors	Symptomatic benign tumors	Malignant primary tumors
		Low-grade tumors	Metastases
			All tumors due to progressive deficits
			All tumors due to cranial nerve deficits or endocrine deficiency
			Posterior fossa tumors
Spine	Degenerative spinal diseases except for neurologic deficits	Benign tumors which are asymptomatic and progress slowly	All spinal diseases with acute motor deficits Unstable fractures
		Under-controlled infections	Spinal metastases that are unstable or compressive.
			Progressive spinal infections
Pediatric		Spina bifida occulta	Hydrocephalus
		Lipomyelomeningocele	Shunt infection and dysfunction
		Tethered cord	Myelomeningocele
		Chiari decompression	
		Craniosynostosis	
Cranial trauma			Acute subdural and epidural hematoma
			Chronic subdural hematoma with neurological symptoms
			Depressed or open cranial fracture

In all patients scheduled for surgery, COVID-19 contact histories were asked, clinical evaluations (physical examination, body temperature, respiratory problems, etc.) were performed in terms of COVID-19 symptoms, COVID-19 polymerase chain reaction (COVID-19 PCR) tests were performed using nasopharyngeal swabs, and a chest CT was performed. Each patient scheduled for surgery was informed about the surgical process during the COVID-19 pandemic. In addition to the surgical consent form, written consent was obtained from each patient along with the COVID-19 consent form prepared by the Turkish Neurosurgery Society.^[7]^

In the post-operative period, the patients were followed up for 2 weeks, and for discharged patients, in-home isolation for 2 weeks was implemented. Chest CT and COVID-19 PCR tests were repeated in patients who developed symptoms during this period.

If at least one of the tests (chest CT and COVID-19 PCR) performed 2 weeks before and after surgery indicated COVID-19, the patient was evaluated as COVID-19-positive.

### Protection of Health-care Personnel

In the current Chinese guidelines for aerosol-transmitted diseases, measures for healthcare workers are currently divided into three levels: Level 1 being the lowest prevention level and level 3 being the highest prevention level (Table 2).^[8]^

**Table 2.** COVID-19 protective equipment for medical staff^[8]^

	**Equipment of Protection**
Level 1	Disposable surgical cap
	Disposable surgical mask
	Work uniform
	Disposable latex gloves and disposable isolation clothing if necessary
Level 2	Disposable surgical cap
	Medical protective mask (N95)
	Work uniform
	Disposable medical protective. Uniform
	Disposable latex gloves
	Goggles
Level 3	Disposable surgical cap
	Medical protective mask (N95)
	Work uniform
	Disposable medical protective uniform
	Disposable latex gloves
	Full-face respiratory protective devices or powered air-purifying respirator

COVID-19-negative outpatient and inpatient measures were implemented under level 2 protections. Surgical interventions were performed under level 2 protection. Level 3 protection was implemented in COVID-19-positive or suspicious cases or in cases requiring urgent intervention.

### Study Population

The records of the patients who underwent surgery in our clinic were retrospectively reviewed in the neurosurgery department of Sisli Hamidiye Etfal Training and Research Hospital between March 2019 to July 2019 and March 2020 to July 2020 when the COVID-19 pandemic occurred in our country.

### Patient Selection

Three hundred and seventeen patients had undergone surgical procedures in our clinic between March 2019 and July 2019; however, from this group, only 136 patients operated by the same team were included in this study in the event that they would be operated if they were referred during the COVID-19 pandemic and were classified into group N. One hundred and eighty-one patients who underwent elective spinal surgeries or had cranial pathologies that do not cause harm when surgery is delayed. As a result, 66 patients who met the specified criteria and underwent surgery during the COVID-19 pandemic between March 2020 and July 2020 were included in this study and were classified into Group P.

### Data Collection

For the comorbidity data, the criteria considered by the American College of Surgeons National Surgical Quality Improvement Program 11 surgical risk calculator 12 were used. Patient characteristics including age, sex, type of surgery, trauma history, ventilator dependency, disseminated cancer, the presence of COVID-19, additional diseases, and anesthesia type were recorded (Table 3). While examining the complications, infection, surgery, and anesthesia duration, hospital stay, ICU stay, and discharge situation (Karnofsky Performance Status Scale score, Glasgow Coma Scale score, and exitus) were recorded, and the Clavien-Dindo classification was used (Table 4).^[9,10]^

**Table 3.** Patient demographics

	**Total (n=202)**		**2019 (n=136)**		**2020 (n=66)**		**P**
	**n**	**(%)**	**n**	**(%)**	**n**	**(%)**	
Age, years	47.9±19.5		48.0±20.3		47.7±18.0		0.912
	51 (0–92)		49.5 (0–92)		51.5 (1–75)		
Age group, years							
<65 years	165	81.7	110	80.9	55	83.3	0.314
65–74 years	25	12.4	15	11.0	10	15.2	
75–84 years	8	4.0	7	5.1	1	1.5	
>85 years	4	2.0	4	2.9	0	0.0	
Sex							
Male	124	61.4	84	61.8	40	60.6	0.874
Female	78	38.6	52	38.2	26	39.4	
Type of surgery							
Elective	144	71.3	109	80.1	35	53.0	<0.001
Urgent	58	28.7	27	19.9	31	47.0	
Trauma							
No	181	89.6	120	88.2	61	92.4	0.360
Yes	21	10.4	16	11.8	5	7.6	
Ventilator dependency							
No	177	87.6	125	91.9	52	78.8	0.025
Yes, Pre-operative	5	2.5	2	1.5	3	4.5	
Yes, Post-operative	20	9.9	9	6.6	11	16.7	
Disseminated cancer							
Yes	7	3.5	7	5.1	0	0.0	0.098
No	195	96.5	129	94.9	66	100	
Additional disease							
Yes	64	31.7	40	30.1	23	34.8	0.501
Hypertension requiring medication	21	10.4	14	10.3	7	10.6	0.946
Diabetes	27	13.4	14	10.3	13	19.7	0.065
Asthma	8	4.0	3	2.2	5	7.6	0.117
Cancer	8	4.0	8	5.9	0	0.0	0.055
History of severe COPD	6	3.0	2	1.5	4	6.1	0.090
Other	7	3.5	4	2.9	3	4.5	0.685
Anesthesia							
General	183	90.6	117	86.0	66	100.0	0.001
Local	19	9.4	19	14.0	0	0.0	

**Table 4.** Complications and outcomes

	**Total (n=202)**		**2019 (n=136)**		**2020 (n=66)**		**p**
	**n**	**%**	**n**	**%**	**n**	**%**	
Complications, all	32	15.8	14	10.3	18	27.3	0.002
Thrombotic complications	2	1.0	0	0.0	2	3.1	0.103
Hemorrhagic complications	1	0.5	1	0.7	0	0.0	1.000
Pulmonary complications	13	6.5	2	1.5	11	16.9	<0.001
Cardiac complications	0	0.0	0	0.0	0	0.0	-
Neurological complications	14	7.0	9	6.6	5	7.7	0.773
Local complications	10	5.0	6	4.4	4	6.2	0.730
Infection	23	11.4	5	3.7	18	27.3	<0.001
Antibiotic use	23	11.4	5	3.7	18	27.3	<0.001
Operation duration (surgery + anesthesia), min	220.3±77.2		210.0±84.7		241.4±53.3		0.014
	240 (35–350)		240 (35–350)		250 (45–330)		
Hospital stay, day	7.9±15.0		7.1±15.8		9.6±13.0		0.093
	4 (1–168)		4 (1–168)		4 (1–77)		
Intensive care unit stay, day	3.2±11.7		2.8±12.9		4.1±8.5		0.023
	1 (0–146)		1 (0–146)		1 (0–41)		
Discharge Karnofsky Score	87.9±18.5		87.0±17.7		89.8±20.0		0.062
	100 (20–100)		100 (30–100)		100 (20–100)		
Discharge Glasgow Come Scale	14.5±1.4		14.6±1.1		14.4±1.9		0.522
	15 (3–15)		15 (9–15)		15 (3–15)		
Exitus	3	1.5	1	0.7	2	3.0	0.249

### Statistical Analysis

Statistical Package for the Social Sciences for Windows (version 15.0; IBM Corporation, Armonk, NY, USA) was used for statistical analysis. Descriptive statistics were presented as numbers and percentages for categorical variables and mean, standard deviation, minimum, maximum, and median for numerical variables. The numerical variables in two independent groups were compared using the Mann-Whitney U test since the condition of normal distribution was not met. The rates in the groups were analyzed using the Chi-square test. P<0.05 was used to denote statistical significance.

## Results

No significant difference in the mean age, age distribution, and gender ratio between the two groups (p=0.874, p=0.912, and p=0.314, respectively). The rate of emergency surgeries in Group P was significantly higher than that in Group N (p<0.001). A statistically significant difference in the proportion of ventilator-dependent patients was found between the two groups (p=0.025). The proportion of postoperative ventilator-dependent patients was higher in Group P than that in Group N. In Group P, all patients received general anesthesia (Table 3). The total number of surgeries decreased by 79% in 2020 compared with that in 2019 (2019: n, 317; 2020: n, 66). Moreover, the number of spinal and cranial trauma cases decreased by 68% in 2020 compared with that in 2019 (2019: n, 16; 2020: n, 5).

The total operation time and mean surgical time in Group P were significantly higher than those in group N (p=0.014 and p=0.023, respectively). The incidence rate of complications in Group P was statistically significantly higher than that in Group N (p=0.002). Moreover, among all complications, the rate of pulmonary complications was statistically significantly higher in Group P than that in Group N (p<0.001).

The averages of infection, antibiotic use, and ICU hospitalization (days) in Group P were statistically significantly higher than those in group N (p<0.001, p<0.001, and p=0.023, respectively). Moreover, 10 of 11 patients who developed pulmonary complications in group P were in the COVID-19-positive group (Table 4). Of the 14 COVID-19-positive patients, only four did not develop pulmonary complications (Table 5).

**Table 5.** Complications in COVID-19-positive patients

		**COVID-19 positive (n=14)**
	**n**	**%**
Complications, all	11	78.5
Thrombotic complications	1	7.1
Hemorrhagic complications	0	0.0
Pulmonary complications	10	71.4
Cardiac complications	0	0.0
Neurological complications	1	7.1
Local complications	1	7.1
Exitus	1	7.1

## Discussion

COVID-19 was declared as an international public health emergency by the WHO on January 30, 2020.^[1]^ COVID-19, which has spread rapidly after its detection in Wuhan, China in December 2019, has affected more than 79 million individuals worldwide and caused more than 1.6 million deaths.^[2]^

In a global study investigating, the impact of COVID-19 on neurosurgery practice involving 494 participants from sixty countries, participants agreed that rapidly progressive neuro-oncological cases and unstable vascular cases are not urgent; however, their delay carries a high risk. Among all respondents, 53% have reported that all elective cases were canceled and clinics were closed. Moreover, 46% of the total participants and 55% of participants in countries more affected by the pandemic have reported that the intensity of surgeries decreased by more than 50%.^[11]^ In addition, the 79% reduction in the total number of surgeries in this study supports these data.

Legal bans and home-stay strategies during the pandemic significantly reduced the number of individuals affected by spinal and cranial trauma and enabled health-care teams to focus on patients with COVID-19.^[12,13]^ Our clinic is one of the centers where trauma patients are referred from neighboring hospitals during the pandemic. While 16 trauma-related emergency operations were performed in our clinic in 2019 (one cranial depressed fracture, three epidural hematoma, four acute subdural hematoma, four chronic subdural hematomas with trauma history, and four spinal fracture cases), five emergency surgeries were performed due to trauma during the COVID-19 pandemic (two acute subdural hematoma and three spinal fracture cases). Trauma patients who presented to centers where surgeries were stopped completely during the pandemic were referred to our clinic. Despite this, the number of spinal and cranial trauma cases decreased in 2020 compared with that in the previous year in this study.

In 2019, 159 patients had elective surgery for spinal degenerative disease; however, these patients were excluded from this study where complications were compared. However, there is still a remarkable situation that these diseases continue during the COVID-19 period. In July 2020, when polyclinics started to work normally in our country, the number of patients applying to the neurosurgery clinic due to spinal degenerative diseases has increased. Four hundred and eighty (76%) of 723 patients who applied to the neurosurgery outpatient clinic in July 2020 presented with spinal complaints. One hundred and seventy-three (36%) of 480 patients who presented with spinal complaints had spinal degenerative diseases. Based on the anamnesis from these patients, patients were not admitted to the hospital during the pandemic because they viewed hospitals as high risk places for infection and the health ministry’s warnings in the media. This supported the reason for the decrease in admissions for spinal degenerative diseases predicted by Dobran et al.^[14]^ Considering the risk of the second wave of COVID-19 cases, new regulations should be developed in neurosurgery practice to prevent these patients from suffering from their diseases.

At present, data on complications in patients undergoing surgery during the COVID-19 period are limited. In a study by Lei et al. involving thirty-four patients who underwent surgery during the of COVID-19 incubation period, all patients developed early postoperative pneumonia, 44% required ICU admission, and 20% died.^[15]^ Li et al. have reported 38.5% mortality in 13 patients who underwent elective thoracic surgery.^[16]^

In different clinical studies, low levels of perioperative cerebral oxygen saturation (ScO_2_) are associated with post-operative neurological complications and overall mortality.^[17]^ ScO_2_ level is affected by systemic hemodynamics, systemic oxygen balance, and oxygen delivery.^[18]^ The importance of intensive care and ventilator support in managing patients with COVID-19 is known.^[19]^ In this study, 14 (21%) of the 66 patients in Group P and 11 (8%) of the 136 patients in Group N needed ventilator support. A statistically significant difference in the proportions of ventilator-dependent patients (p=0.025) was found between the two groups. In Group N, preoperatively, two patients required ventilator support and did not develop complications, and complications were observed in two of the nine patients who required post-operative ventilatory support. In Group P, three patients who needed preoperative ventilator support were COVID-19-positive, and one of them developed complications; furthermore, of the 11 patients who required post-operative ventilator support, nine were COVID-19-positive and eight developed complications. The increase in the ventilator dependency can be explained by the COVID-19 pandemic. However, patients who were operated during this period had higher morbidity due to systemic and neurological problems, whose surgery could not be delayed.

In this study, the mortality rate in patients with COVID-19 was lower than those in some studies.^[16,20]^ This can be explained by the fact that 93% of COVID-19-positive patients were under the age of 65 years and had no uncontrollable comorbidities, except for one patient who died. The low post-operative mortality rate in the COVID-19 period can be explained by the fact that our clinic has its own neurosurgery ICU, and thus, surgical patients are allocated a place in the ICU during pre-operative planning, and post-operative ICU and mechanical ventilator support can be provided immediately when necessary. Although the COVID-19-positive patient who died developed pulmonary complications, the clinic of the patient, who was operated urgently due to brainstem compression and hydrocephalus after bleeding of the posterior fossa hemangioblastoma of Von Hippel-Lindau syndrome, caused the high mortality rate, independent of COVID-19.^[21]^ In this study, the rate of complications in Group P was statistically significantly higher than that in group N (p=0.002). In addition, the likelihood of complication occurrence was higher in patients with COVID-19. Eleven (61%) of the 18 patients who developed complications in Group P were COVID-19-positive. Of the 14 COVID-19-positive patients, only three (21%) did not develop complications.

Among the complications, the rate of pulmonary complications was statistically significantly higher in Group P (p<0.001). Only two (14%) of the 14 patients who developed complications in group N had pulmonary complications, whereas 11 (61%) of the 18 patients who developed complications in Group P developed pulmonary complications. Ten (91%) of the 11 patients who developed pulmonary complications in Group P were COVID-19-positive. Of the 14 patients who were COVID-19-positive, only four (29%) did not develop pulmonary complications, and these findings conform to those of other studies.^[15,16,20]^ In line with these data, in the treatment planning of COVID-19-positive patients, surgery should be postponed if possible, and in cases where it cannot be postponed, the patient should be informed that the risk of complications is high, indicating the necessity of precautions and preparations against post-operative risk, especially pulmonary complications.

There are different recommendations for COVID-19-negative patients to protect healthcare personnel in the operating room during the surgical procedure.^[22]^ According to the neuro-oncology team, patients from low-risk areas who have been confirmed to be COVID-19-negative can be operated with level 1 measures.^[22]^ According to the teams of Tongji Medical College, Wuhan, China, and Heinrich-Heine University, Düsseldorf, Germany, healthcare professionals should implement level 2 measures due to the long incubation period of COVID-19.^[22,23]^ In this process, we acted toward taking level 2 measures. COVID-19-positive neurosurgery patients who need urgent surgery have high viral loads.^[23]^ Even if urgent patients do not have clinical symptoms and do not have COVID-19 findings in their CT scans, we have implemented the recommended level 3 measures in performing surgery for COVID-19-positive patients.

The features making this study valuable are that the demographic characteristics of the patient groups in the compared periods and the surgeries performed were similar, the procedures were performed by the same clinic and neurosurgery team, and our clinic is a center to which patients from other hospitals are referred during the pandemic.

In this study, the number of COVID-19-positive patients may be seen as insufficient; however, in this period when the pandemic started for the 1st time in our country, the false-negative rate of PCR tests and clinical uncertainties should be considered. In this study, where complications were evaluated, it should not be forgotten that the surgical team evaluated each patient individually and managed the process very carefully and meticulously. The expected result is a decrease in complications when treated more carefully than normal; however, the opposite was observed in this study. In addition, the risk of complications following surgical procedures will increase during the pandemic where health-care professionals are under intense physical and mental pressure.

The second wave of the pandemic, which is not known for how long it will last, has started to emerge worldwide. The treatment of patients and diseases should continue.

## Conclusion

Considering this study, complication rates may increase, and a higher risk than expected may be encountered. Continuation of treatment of patients should be ensured during the pandemic by taking the necessary precautions and informing the patient about these additional risks.

### Disclosures

**Ethics Committee Approval:** University of Health Sciences, Sisli Hamidiye Etfal Training and Research Hospital, 1899 / 05.25.2021.

**Peer-review:** Externally peer-reviewed.

**Conflict of Interest:** None declared.

**Authorship Contributions:** Concept – M.K., B.S.; Design – B.S., M.K.; Supervision – M.K., B.O; Materials – L.A, E.A; Data collection &/or processing – L.A., E.A.; Analysis and/or interpretation – B.S., S.A.S.; Literature search – B.S., S.A.S.; Writing –B.S., S.A.S.; Critical review – B.Ö., A.Y., A.M.M.
